# *PTEN* genomic deletion predicts prostate cancer recurrence and is associated with low AR expression and transcriptional activity

**DOI:** 10.1186/1471-2407-12-543

**Published:** 2012-11-22

**Authors:** Khalil Choucair, Joshua Ejdelman, Fadi Brimo, Armen Aprikian, Simone Chevalier, Jacques Lapointe

**Affiliations:** 1 Department of Surgery, Division of Urology, McGill University and the Research Institute of the McGill University Health Centre(RI-MUHC), Montreal, H3G 1A4 QC, Canada; 2 Department of Pathology, McGill University and the Research Institute of the McGill University Health Centre(RI-MUHC), Montreal, H3G 1A4, QC, Canada

**Keywords:** Prostate cancer, Prognosis, PTEN, AR

## Abstract

**Background:**

Prostate cancer (PCa), a leading cause of cancer death in North American men, displays a broad range of clinical outcome from relatively indolent to lethal metastatic disease. Several genomic alterations have been identified in PCa which may serve as predictors of progression. *PTEN*, (10q23.3), is a negative regulator of the phosphatidylinositol 3-kinase (PIK3)/AKT survival pathway and a tumor suppressor frequently deleted in PCa. The androgen receptor (AR) signalling pathway is known to play an important role in PCa and its blockade constitutes a commonly used treatment modality. In this study, we assessed the deletion status of *PTEN* along with AR expression levels in 43 primary PCa specimens with clinical follow-up.

**Methods:**

Fluorescence *In Situ* Hybridization (FISH) was done on formalin fixed paraffin embedded (FFPE) PCa samples to examine the deletion status of *PTEN*. AR expression levels were determined using immunohistochemistry (IHC).

**Results:**

Using FISH, we found 18 cases of *PTEN* deletion. Kaplan-Meier analysis showed an association with disease recurrence (*P*=0.03). Concurrently, IHC staining for AR found significantly lower levels of AR expression within those tumors deleted for *PTEN* (*P*<0.05). To validate these observations we interrogated a copy number alteration and gene expression profiling dataset of 64 PCa samples, 17 of which were *PTEN* deleted. We confirmed the predictive value of *PTEN* deletion in disease recurrence (*P*=0.03). *PTEN* deletion was also linked to diminished expression of PTEN (*P*<0.01) and AR (*P*=0.02). Furthermore, gene set enrichment analysis revealed a diminished expression of genes downstream of AR signalling in *PTEN* deleted tumors.

**Conclusions:**

Altogether, our data suggest that *PTEN* deleted tumors expressing low levels of AR may represent a worse prognostic subset of PCa establishing a challenge for therapeutic management.

## Background

Prostate cancer (PCa) strongly affects the male population, and is classified as the most commonly diagnosed cancer and a leading cause of cancer death in North American men [1]. The current prognostic tools, such as pre-operative prostate specific antigen (PSA) levels, histological Gleason grading of biopsy specimens and clinical TNM (tumor, node, metastasis) staging seem unable to accurately risk stratify individual PCa patients at early stages of the disease. Given the wide range of clinical outcomes and the heterogeneity of the disease, the main challenge facing physicians remains to distinguish latent from clinically significant tumors. There is thus a clear need for better prognostic markers.

Androgens are required for maintaining the homeostasis of the normal prostate epithelium. Their effect is mediated via the androgen receptors (AR), a member of the nuclear superfamily of steroid receptor, acting as a transcription factor in prostate cell nuclei. PCa cells have retained the ability to proliferate upon stimulation with androgens, resulting in tumor growth [2]. Thus, PCa patients that experience a recurrence following localized treatment are subjected to androgen deprivation therapy. Although most patients respond well initially to androgen deprivation therapy, almost all of them will eventually experience resistance to treatment and disease progression [3]. Therapeutic options for castrate resistant PCa (CRPC) are limited to chemotherapy regimens that show a modest survival benefit [4]. There is currently no curative treatment for metastatic PCa. Understanding the molecules and the pathways involved in mediating resistance is thus needed for a better clinical management of the disease.

The phosphatidylinositol 3-kinase (PI3K)/AKT signal transduction pathway contributes to cancer growth and survival, and is activated in a broad range of human malignancies including PCa [5]. The phosphatase and tensin homologue deleted on chromosome 10 (*PTEN*) is a tumor suppressor gene on 10q23.3 locus that acts by negatively regulating the PI3K/AKT pathway [6]. In animal models, *PTEN* was shown to be haploinsufficient in tumor suppression [7]. *PTEN* genomic deletion has been detected in human tissues representing all stages of PCa development and progression including High Grade Prostatic Intraepithelial Neoplasia (HGPIN), primary PCa and at higher frequency in metastatic PCa and CRPC [8-15]. Using Fluorescent *in situ* hybridization (FISH), *PTEN* deletion status of primary PCa has been associated with poor outcome [14]. Previous studies in human PCa cell lines and mice models have suggested that inactivation of *PTEN* and PI3K/AKT activation can modulate AR activity and contribute to CRPC [16-18]. These observations provided further rationale to examine PTEN and AR in human prostate tissues.

In this study, we surveyed PCa samples for genomic DNA copy number alterations (CNAs) of the *PTEN* gene using Fluorescent *in situ* hybridization (FISH) and AR expression by immunohistochemistry (IHC). An existing PCa microarray dataset of DNA CNAs by array comparative genomic hybridization (CGH) and corresponding gene expression profiling were used to validate these findings.

## Methods

### Ethics statement

This study was conducted with the written consent of the participants and approved by the Research Ethics Board of the McGill University Health Centre (study BMD-10-115).

### Tissue samples

Formalin fixed paraffin embedded (FFPE) blocks (n = 43) of primary tumors and adjacent benign tissues from radical prostatectomy were retrieved from the Department of Pathology. Duplicate tissue cores (1mm diameter) were assembled into tissue microarrays (TMAs). Haematoxylin and eosin (H&E)-stained TMA sections were reviewed to determine the presence of representative areas of the original samples. The clinicopathologic features of the cohort are summarized in Table 1. Recurrence-free interval was defined as the time between date of surgery and the date of first PSA increase >0.2ng/ml or the date of last follow-up without PSA increase (censored). Kaplan-Meier survival analysis (log-rank test) was performed using WinStat (R. Fitch Software).

**Table 1:** Clinicopathologic parameters of the study subjects

	**n=43**
**Median age** (**range**, **years**)	63 (47–76)
**Median follow**-**up** (**months**)	62
**Median PSA at surgery** (**ng**.**ml**-**1**)	8.7
**Biochemical recurrence**	12 (28%)
**Gleason score**	
≤6	13 (30%)
=7	23 (54%)
≥8	7 (16%)
**Pathological stage**	
≤T2	27 (63%)
≥T3	16 (37%)

### Fluorescent *in situ* hybridization (FISH)

Dual-color FISH was carried out on TMA sections using the BAC clone RP11-383D9 (BACPAC Resources Center, Oakland, CA) mapping to the *PTEN* gene on chromosome 10q23.3 region and the commercially available CEP10 Spectrum Green probe (CEP 10, Abbott Molecular, Abbott Park, IL), which spans the 10p11.1-q11.1 centromeric region. RP11-383D9 DNA was labeled with Spectrum Orange-dUTP (Enzo Life Science, Farmingdale, NY) using the Nick Translation Reagent Kit (Abbott Molecular). The 5 μm TMAs sections were de-paraffinized in 6 changes of xylene before immersion in 95% ethanol. The slides were then placed in 0.2 N HCl solution at room temperature (RT°) for 20 min followed by a 2-hour incubation at 80°C in 10 mM citric acid buffer (pH 6) for pre-treatment. Specimens were digested in 0.1 mg/ml protease I (Abbott Molecular), and then fixed for 10 min in formalin before dehydration in an ethanol series. The two probes and target DNA were co-denatured at 73°C for 6 min and left to hybridize at 37°C O/N using the ThermoBrite system (Abbott Molecular). Post-hybridization washes were performed in 2xSSC and 0.3% NP40/0.4xSSC at 73°C for 2 min and 1 min respectively, followed by a 30 sec incubation at RT° in 2xSSC.

### FISH data analysis

In order to evaluate the 10q23.3 copy number, we counted fluorescent signals in 100 non-overlapping interphase nuclei for each sample. 4',6-Diamidino-2-phenylindole (*DAPI III*, Abbott Molecular) staining of nuclei with reference to the corresponding H&E-stained tissue identified the areas of adenocarcinoma. Using hybridization in 30 benign control cores, 10q23.3 deletion was defined as ≥15% (mean + 3 standard deviation in non-neoplastic controls as described [19,20]) of tumor nuclei containing one or no 10q23.3 locus signal and by the presence of two CEP10 signals. Images were acquired with an Olympus IX-81 inverted microscope at 96X magnification using ImageProPlus 7.0 software (MediaCybernetics, Rockville, MD).

### Immunohistochemistry (IHC) staining

Immunostaining of AR on TMAs sections was performed using a mouse anti-AR antibody (N-terminal AR 441, NeoMarker, Fremont, CA) and the Envision detection kit (Dako, Carpinteria, CA). The 5 μm TMAs sections were de-paraffinized in a series of xylene and hydrated in a graded series ethanol solutions. Heat-induced antigen retrieval was performed by immersing the slides in 10 mM citric acid buffer solution (pH 6) and boiling for 30 min using microwave energy. The slides were left in solution to cool down for 30 min at room temperature. Endogenous peroxydase activity was blocked for 5 minutes (Dako). After a 60 min block with 10% normal goat serum in PBS (Dako), the primary antibody (1:50 dilution in Dako antibody diluent) was used for two hours at room temperature. Chromogenic detection was carried out using a peroxidase-conjugated secondary antibody (30 min) and DAB reagents (10 min) provided with the Envision detection kit. Tissue sections were counterstained with Meyer’s Haematoxylin (Thermo Scientific, Waltham, MA).

### IHC data analysis

Nuclear staining was assessed by two independent observers using the H-score method described in [21,22]. Briefly, H-score was obtained by computing the product of staining intensity (i=0-3) and the proportion of cells with the specific intensity (0–100), in areas of adenocarcinoma as identified with reference to the corresponding H&E-stained tissue. The H-scores were adjusted to give the highest score a value of 100. AR H-scores were compared between *PTEN* deleted and non deleted specimens categories with the Mann–Whitney *U*-Test ( http://elegans.som.vcu.edu/~leon/stats/utest.html).

### Gene set enrichment analysis (GSEA)

Analysis [23] was performed using GSEA software version 2.07 (Broad Institute, Cambridge, MA) with the previously published gene expression data of 64 prostate tumors by Lapointe et al. [24] stratified by their *PTEN* genomic status as reported in the corresponding array CGH study [9]. Two androgen-responsive gene sets (R1881-treated LNCaP cells) were tested for enrichment in the gene expression microarray data: a curated set of 82 genes (NELSON_RESPONSE_TO_ANDROGEN_UP, [25]) from the Molecular Signatures database (MSigDB, C2) and a set of 207 genes reported by DePrimo et al. [26]. Lapointe et al gene expression study used for GSEA included data for respectively 71 and 204 genes of Nelson et al. and DePrimo et al. androgen-responsive gene sets. A thousand permutations were done and the false discovery rate (FDR) was estimated.

## Results

### FISH analysis and *PTEN* deletion status

**Figure 1:**
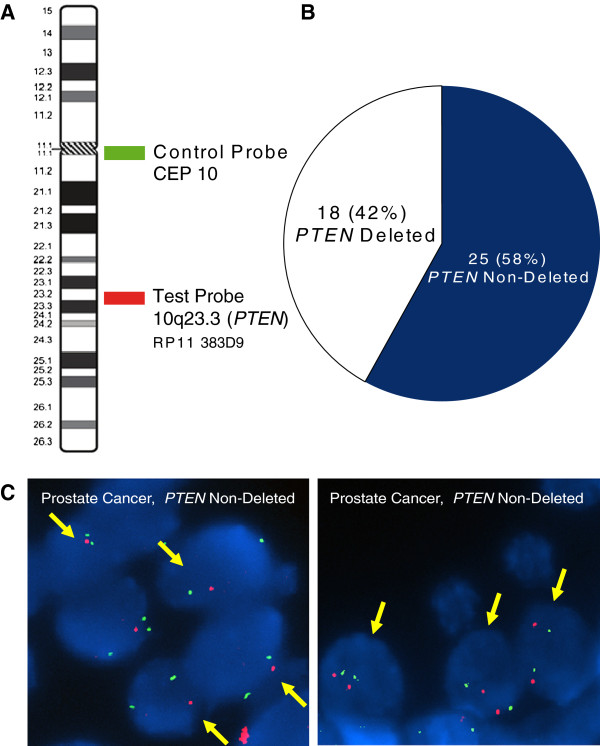
**Dual color FISH analysis of *PTEN *deletion in primary PCa.A**) BAC DNA mapping to chromosome 10q23.3 (*PTEN*) was fluorescently labelled and co-hybridized with fluorescent Centromere 10 control probe to detect *PTEN* deletion in tumor samples. **B**) *PTEN* deletion status of 43 primary PCa samples determined by FISH. **C**) FISH for *PTEN* status in representative interphase nuclei of prostate samples. On the left panel, the FISH image shows 1 red signal (10q23.3 locus) and two green signals (centromere 10) per nuclei indicating a *PTEN* deletion. On the right panel, the FISH image shows two red signals and two green signals in the nuclei indicating no *PTEN* deletion.

**Figure 2:**
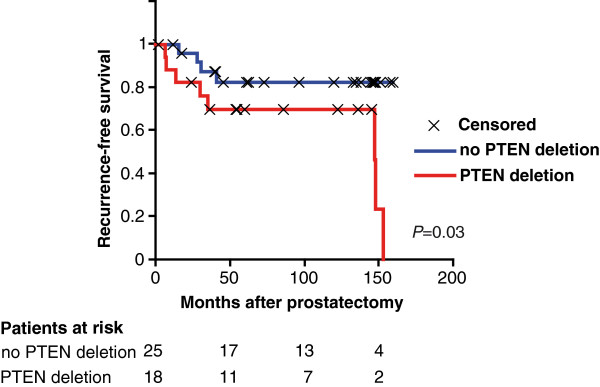
**Prognostic value of *PTEN *deletion in PCa.** Kaplan-Meier recurrence-free survival analysis based on *PTEN* deletion status determined by FISH (n=43). *P*-value (log-rank test) indicated.

We used FISH to assess the genomic status of *PTEN* at chromosome 10q23.3 on TMAs representing 43 cases of human PCa with clinical follow-up. The clinicopathologic characteristics of the study subjects are summarized in Table 1. We found that 18 of 43 tumors harbor a hemizygous deletion of *PTEN* (Figure 1). No homozygous deletion was detected in these samples. We did not find any significant association between *PTEN* status and tumor Gleason score, surgical stage, and preoperative PSA levels of patients (data not shown). To further evaluate the clinical significance of the *PTEN* deletion, we stratified the 43 cases based on their *PTEN* deletion status and performed a Kaplan-Meier survival analysis. Figure 2 shows that *PTEN* deletion was associated with a significant shorter time to recurrence (P=0.03).

### IHC and AR expression

**Figure 3:**
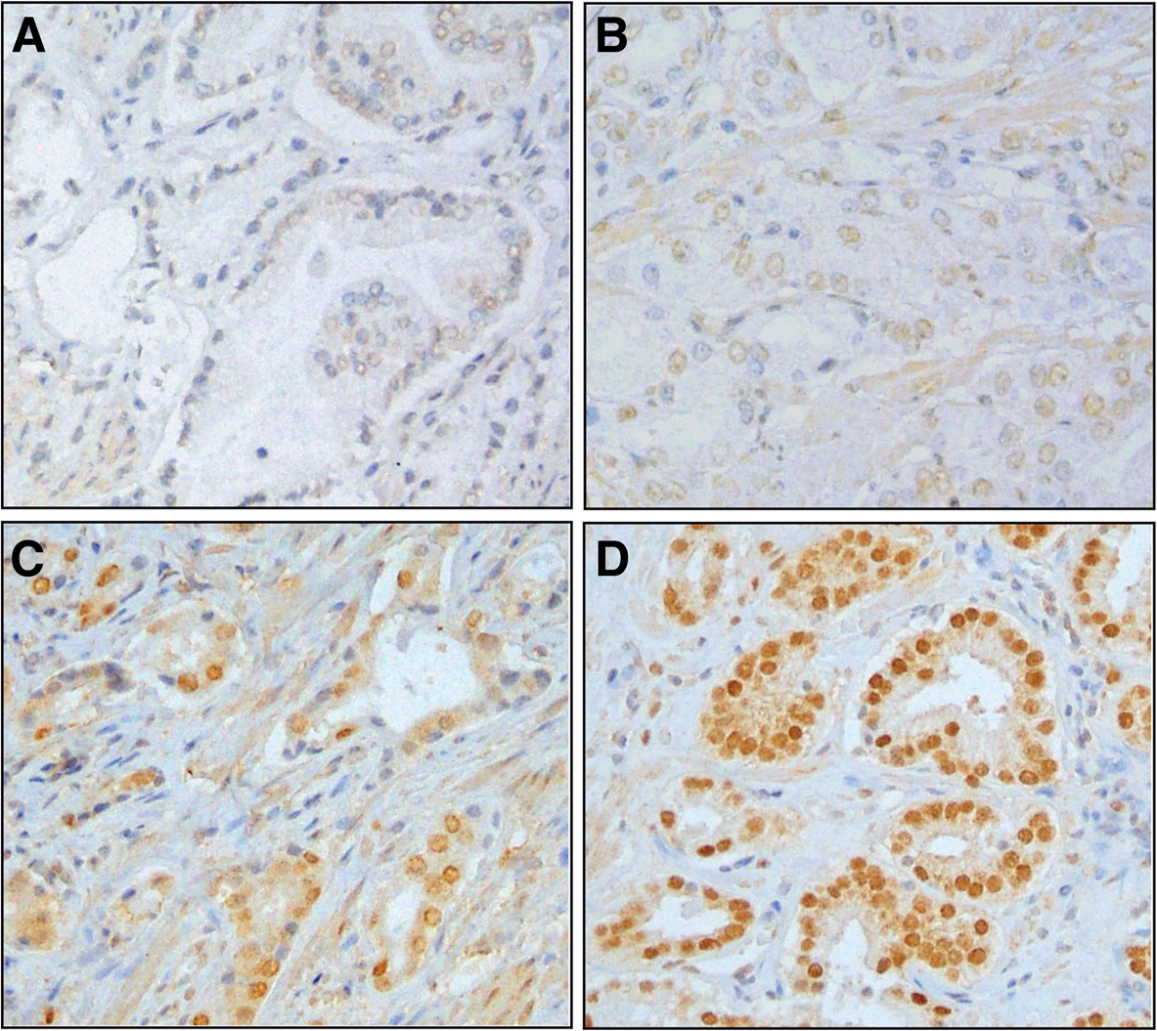
**AR IHC staining of PCa.** Examples of nuclear AR staining corresponding to the variation of the adjusted H-score scale with **A**=10, **B**=28, **C**=71 and **D**=76. Original magnification 400X.

**Figure 4:**
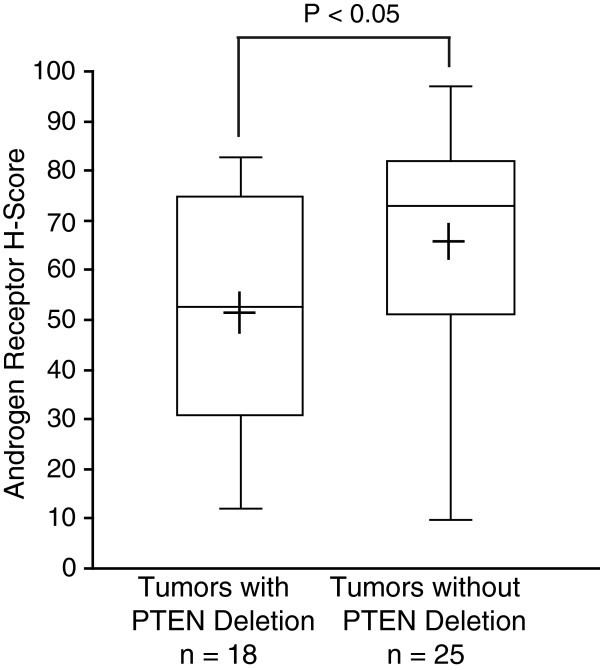
***PTEN *deletion is associated with low AR expression in PCa.** Adjusted H-score of nuclear AR (IHC) was compared between PCa with and without *PTEN* deletion determined by FISH. The box-plot shows the mean (+ sign), the 25th, 50th (median), 75th percentiles of AR H-score including the minimum and maximum (two-sided Mann–Whitney *U*-test, *P*-value indicated).

We used the same set of tumors to estimate the levels of nuclear AR expression by IHC. For each sample, the H-score was calculated to take into account the proportion of stained cells on the TMA cores as well as the intensity of the nuclear staining. The range of the H-score adjusted to 100 varied across the samples from 10 to 100 (median=70, n=43). AR immunostaining of specimens with different H-scores are shown as examples in Figure 3. In these samples, AR immunostaining was not significantly associated with the Gleason score, surgical stage, preoperative PSA, and recurrence (not shown). However we found that the AR expression was significantly lower in PCa tumors harboring a *PTEN* deletion compared to those with no deletion of *PTEN* (P<0.05, Figure 4).

### *PTEN* deletion and AR expression in gene expression and DNA copy number alterations dataset

**Figure 5:**
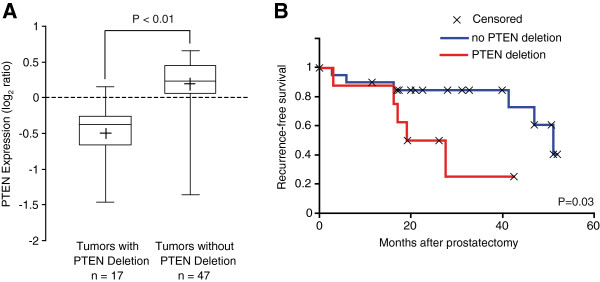
***PTEN *deletion predicts disease recurrence in an independent PCa cohort.A**) In the dataset of Lapointe et al., *PTEN* deletion status of 64 PCa as determined by array CGH was associated with low PTEN mRNA levels measured by gene expression profiling. The box-plot shows the mean (+ sign), the 25th, 50th (median), 75th percentiles of PTEN expression including the minimum and maximum (unequal variance *t*-test, *P*-Value indicated). Values are reported as log2 ratios, normalized to the sample-set mean. **B**) Kaplan-Meier analysis of recurrence-free survival based on *PTEN* deletion status of a subset of the PCa cohort for which the clinical follow-up was available (n=29). *P*-value (log-rank test) indicated.

**Figure 6:**
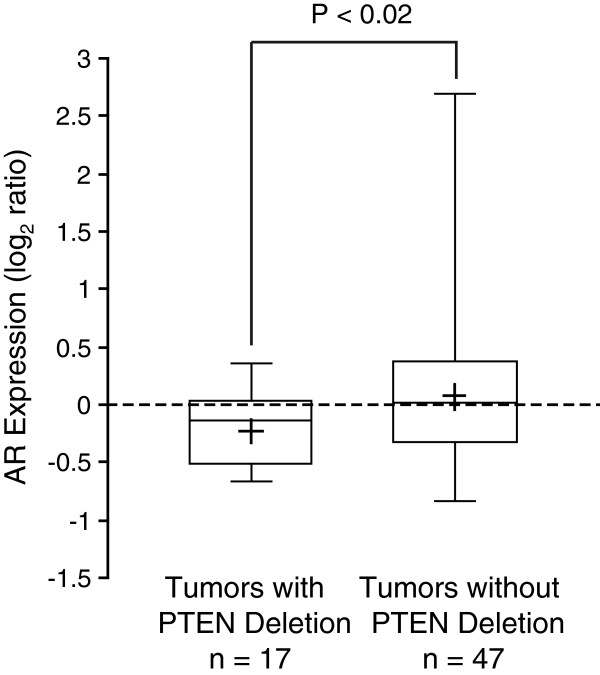
***PTEN *deletion is associated with low AR expression in an independent PCa cohort.***PTEN* deletion status as determined by array CGH was associated with low AR mRNA levels as measured by gene expression profiling of 64 PCa cases from the dataset of Lapointe et al. The box-plot shows the mean (+ sign), the 25th, 50th (median), 75th percentiles of AR expression including the minimum and maximum (unequal variance *t*-test, *P*-value indicated). Values are reported as log_2_ ratios, normalized to the sample-set mean.

To confirm our observations, we examined a previously published PCa gene expression study [24], for which the *PTEN* deletion status was assessed by array CGH [9]. This independent data set of 64 PCa samples included 29 cases with clinical follow-up. We found that 17 of 64 tumors harbor a deletion of *PTEN* which was significantly associated with a reduced levels of PTEN mRNA (*P*<0.01, Figure 5A). A Kaplan-Meier survival analysis performed on the 29 cases with clinical follow-up revealed that the *PTEN* deletion was associated with early disease recurrence (*P*=0.03, Figure 5B). The *PTEN* deletion was also associated with a reduced levels of AR mRNA (*P*=0.02, Figure 6). However, AR mRNA levels did not predict biochemical recurrence in these samples (data not shown).

### Androgen-regulated genes and *PTEN* deletion

**Figure 7:**
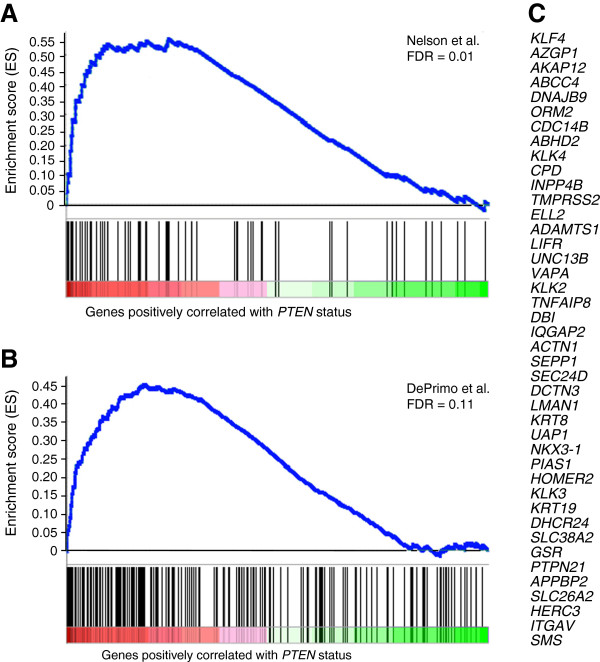
***PTEN *status is associated with AR signalling.** GSEA was performed with previously published gene expression data of 64 prostate tumors (Lapointe et al.) stratified by their *PTEN* genomic status. Two androgen responsive gene sets were tested: **A**) a curated set of 71 genes (NELSON_RESPONSE_TO_ANDROGEN_UP, Nelson et al.) from the Molecular Signatures database (MSigDB, C2) and **B**) a set of 204 genes reported by DePrimo et al. GSEA identified enrichment of androgen responsive genes in *PTEN* positive samples. The enrichment score (ES, y-axis) reflects the degree to which an androgen responsive gene set is overrepresented at the top ranked list of genes according to the *PTEN* status (ranked in descending order from left to right, x-axis). Enrichment is evidenced by the early positive deflection of the running sum curve (blue line). A thousand permutations were done and the false discovery rate estimated (FDR). **C**) Genes from Nelson et al. that contribute to the enrichment core.

To assess whether the reduced AR levels of expression observed in *PTEN* deleted tumors had consequences on AR signalling, we performed GSEA on the microarray data of the 64 PCa stratified by their *PTEN* genomic status. GSEA is a computational method that determines whether an *a priori* defined set of genes shows statistically significant, concordant differences between two phenotypes [23], in our case the *PTEN* status. We first tested a curated gene set from the molecular signature database (MSigDB,C2) identified as NELSON_RESPONSE_TO_ANDROGEN_UP, [25]. The plot in Figure 7A shows the significant enrichment of the AR-regulated genes in tumors with no deletion of *PTEN* compared to those with a deletion (FDR of 0.01). To further confirm this result, we tested a second set of androgen regulated genes reported by DePrimo et al. [26] and found also an enrichment of expression of these genes in tumors with no *PTEN* deletion (FDR=0.13, Figure 7B). Genes from Nelson et al. that significantly contribute to the enrichment core are shown in Figure 7C.

## Discussion

In this study, we have shown in two independent sets of PCa samples that the *PTEN* genomic deletion was associated with early disease recurrence and reduced levels of AR expression. In microarray gene expression data, the *PTEN* deletion was also associated with a down regulation of AR-driven genes.

The frequency of *PTEN* deletion in our FISH study (40%) is within the range of previous reports [8,10,12,14,15]. Our survival analysis further confirms the association of *PTEN* genomic deletion and poor outcome of PCa reported earlier [14] and its potential use as a prognostic marker. Clinical relevance is also supported by the recent literature detecting *PTEN* deletion at high frequency in CRPC samples [11], in circulating tumor cells [27] and its association with PCa death [11,28]. Further validation in larger cohorts would be critical to compare its predictive value with the current prognostication tools.

The intriguing finding of our study was the reduced levels of AR expression quantified by H-score in tumors harboring a *PTEN* deletion. We found a similar association between *PTEN* deletion and AR transcript levels in a PCa microarray dataset. The differential expression of AR according to the *PTEN* tumor status has not been well documented so far. A pilot IHC study has found a positive correlation between AR and PTEN expression [29]. In contrast, Sircar et al. reported a positive correlation between *PTEN* deletion status and AR expression [11] in CRPC samples. These results likely reflect two different stages of the disease: CRPC and untreated PCa. The genomic amplification of AR is known to occur in CRPC but rarely in untreated PCa [30], thereby explaining differences in results.

Previous *in vitro* studies in cell lines derived from advanced PCa suggested that PTEN could act as suppressor of AR activity [31,32]. It was also reported that the activation of PI3K/AKT pathway can suppress the AR activity in low passage LNCaP and enhance AR activity in high passage, hence suggesting modulation as cells evolve towards less responsive status [33]. In models representing less advanced disease, re-expression of PTEN in *PTEN* null murine cells did not affect AR expression, but upregulated the AR transcriptional activity [34]. Another group reported that *PTEN* null murine prostate cells had a reduced AR protein levels compared to wild-type *PTEN* cells and the AR protein levels were partly restored by the PI3K/mTOR inhibitor BEZ235 [35]. The latter observation would suggest that the activation of PI3K pathway may in part explain the reduced AR levels in *PTEN* deleted tumors. A shown by Lin et al., it is also possible that PTEN interacts directly with AR and promotes its degradation [31]. Underlying mechanisms of how *PTEN* deletion in human tumors is associated with lower AR expression and transcriptional activity need to be further explored.

Given their reduced levels of AR expression, the *PTEN* deleted tumor cells are expected to be less responsive to androgen ablation treatment. In support of this hypothesis, it was reported that CRPC and early biochemical recurrence were associated with reduced immunoreactivity of PTEN and AR in the PCa samples harvested before treatment initiation [29]. The addition of an inhibitor of PI3K/mTOR to the standard androgen ablation treatment of advanced PCa may therefore be beneficial to patients with *PTEN* deleted tumor.

Some previous studies have found that low levels of AR were associated with PCa recurrence [36,37] while others reported the opposite [38,39]. In our study, AR levels of expression were not significantly associated with PCa recurrence. The antibody used, IHC technique and scoring methods may explain the differences in the findings. Given the limited number of patients of our study, a detailed analysis of AR and PTEN in a large cohort of patients with follow-up is warranted.

During the course of our study, two groups also showed a reduced expression of androgen regulated genes in human *PTEN* deleted PCa by microarray analysis [34,35]. In our analysis, the androgen regulated genes enriched in tumor with no deletion of *PTEN* include genes expressed in normal prostate luminal epithelium such as KLK3 (PSA), TMPRSS2, and NKX3-1. Of interest, the list includes AZGP1 previously reported as a surrogate marker for subtype-1 tumors, a favourable prognostic subclass of PCa defined by gene expression pattern analysis [24]. AZGP1 prognostic value was further confirmed by two other studies [40,41]. Previous GSEA has also revealed enrichment of androgen-responsive genes in subtype-1 tumors [42]. Consistant with our findings, the confirmation of intact *PTEN* status in subtype-1 tumors from the array CGH data may, at least in part, explain their androgen-regulated gene expression feature and good clinical outcome.

## Conclusions

Although limited by the small sample size of this study, our preliminary data support that *PTEN* deletion is associated with PCa recurrence and may thus serve as prognostic marker. As proposed, the low expression of AR and its target genes associated with *PTEN* deletion may have consequences on response to androgen ablation therapy and may be an indication for the introduction of additional therapeutic modalities.

## Competing interests

The authors declare that they have no competing interests.

## Authors' contributions

Conceived and designed the experiments: KC, JE, JL. Performed the experiments: KC, JE. Analyzed the data: KC, JE, FB, JL. Contributed materials/clinical data: AA. Wrote the paper: KC, JE, SC, JL. All authors read and approved the final manuscript.

## Pre-publication history

The pre-publication history for this paper can be accessed here:

http://www.biomedcentral.com/1471-2407/12/543/prepub
